# Effects of reductive soil disinfestation combined with different types of organic materials on the microbial community and functions

**DOI:** 10.1128/spectrum.00802-23

**Published:** 2024-01-17

**Authors:** Yu Zhan, Yi Zhou, Ergang Wang, Xinyue Miao, Tingting Zhou, Ning Yan, Changbao Chen, Qiong Li

**Affiliations:** 1 Jilin Ginseng Academy, Changchun University of Chinese Medicine, Changchun, China; 2 School of Pharmaceutical Sciences, Changchun University of Chinese Medicine, Changchun, China; Huazhong Agricultural University, Wuhan, China

**Keywords:** reductive soil disinfestation, soil degradation, organic amendments combination, microbial community, microbial functional

## Abstract

Reductive soil disinfestation (RSD) is an effective method to inhibit soilborne pathogens. However, it remains unclear how RSD combined with different types of organic materials affects the soil ecosystems of perennial plants. Pot experiments were conducted to investigate the effects of RSD incorporated with perilla (PF), alfalfa (MS), ethanol, and acetic acid on soil properties, enzyme activities, microbial communities and functions, and seedling growth. Results showed that RSD-related treatments improved soil properties and enzyme activities, changed microbial community composition and structure, enhanced microbial interactions and functions, and facilitated seedling growth. Compared with CK, RSD-related treatments increased soil pH, available nitrogen, and available potassium contents, sucrase and catalase activities, and decreased soil electric conductivity values. Meanwhile, RSD-related treatment also significantly reduced the relative abundance of *Fusarium* while increasing the relative abundance of *Arthrobacter*, *Terrabacter*, and *Gemmatimonas*. The reduction was more evident in PF and MS treatment, suggesting the potential for RSD combined with solid agricultural wastes to suppress pathogens. Furthermore, the microbial network of RSD-related treatment was more complex and interconnected, and the functions related to carbon, nitrogen, sulfur, and hydrogen cycling were significantly increased, while the functions of bacterial and fungal plant pathogens were decreased. Importantly, RSD-related treatments also significantly promoted seed germination and seedling growth. In summary, RSD combined with solid agricultural wastes is better than liquid easily degradable compounds by regulating the composition and function of microbial communities to improve soil quality and promote plant growth.

**IMPORTANCE:**

Reductive soil disinfestation (RSD) is an effective agricultural practice. We found that RSD combined with solid agricultural wastes is better than that of liquid easily degradable compounds, may improve soil quality and microbial community structure, inhibit the proliferation of pathogenic bacteria, and contribute to the growth of replanted crops. Thus, RSD combined with solid agricultural wastes is more effective than liquid easily degradable compounds.

## INTRODUCTION

Due to economic interests and land resource shortages, highly intensive agriculture has become prevalent in the contemporary world (1). Unfortunately, continuous monoculture and over-fertilization frequently jeopardize highly intensive agricultural production systems, which inevitably lead to soil degradation and soilborne disease outbreaks (2–4). Severe soilborne diseases have become a worldwide problem, causing considerable economic losses and sabotaging agricultural sustainability (5, 6). According to statistics, more than 70% of medicinal plants have the above problems, such as *Panax ginseng* C. A. Mey. (7), *Salvia miltiorrhiza* Bunge (8), *Panax notoginseng* (Burk.) F. H. Chen (9), which seriously limit the growth of medicinal plants and cause a lot of economic losses. Therefore, it is imperative to find an efficient method for maintaining the medicinal plants’ sustainable development under highly intensive production systems.

Over the last century, soil microbiologists have developed a variety of methods to combat soilborne diseases (10). A pre-planting soil management practice called reductive soil disinfestation (RSD) was independently developed in 2000 by scientists from Japan and the Netherlands (11, 12). RSD creates anaerobic soil conditions by applying easily decomposed organic materials to the soil, adding water for irrigation, and covering plastic films, which soil microorganisms change from “aerobic” to “anaerobic,” and produce organic acids, hydrogen sulfide, ammonia, and other substances to inhibit the growth of soilborne pathogens (13, 14). A number of soilborne plant pathogens have been demonstrated to be inactivated by RSD treatments, including Fusarium wilt (14), verticillium wilt (15), and damping-off disease (16). Meanwhile, RSD practice can also restore soil degradation caused by acidification and secondary salinization (17). Medicinal plants are prone to failure when repeatedly replanted in the same soil due to changes in the soil microbial community, accumulation of self-toxic substances, nutritional imbalances, and deterioration of physicochemical properties (9, 14). It is thus possible to reduce medicinal plant replanting failure by implementing RSD practices.

Different types of organic materials can have different effects on RSD practices (18). Currently, organic materials are mainly divided into two categories: liquid and easily degradable materials (like molasses and ethanol) and solid agricultural wastes (like crop straw and livestock manure) (19, 20). A study discovered that using wheat bran in RSD treatment could effectively inhibit spinach fusarium wilt, and the incidence could be reduced to 21.1% (21). Additionally, molasses is often applied in combination with livestock and poultry manure as a carbon source for RSD practice to inhibit fungi and nematodes (22, 23). This is because different types of organic materials can stimulate the reorganization of different microbiota through anaerobic degradation, especially the core microbiota, whose antagonism, competition, and parasitism can better inhibit pathogenic bacteria (24). Meanwhile, soil microorganisms play a crucial role in soil ecosystems, serving a variety of functions including controlling diseases, retaining nutrients, decomposing organic matter, and forming humus (25, 26). It is possible to improve the effectiveness of RSD practices by using organic materials. Nevertheless, it remains unclear how different types of organic materials will affect soil ecosystem services provided by the soil microbial community. Thus, it is particularly imperative to explore the selection of organic materials during the RSD process in order to assess changes in soil microbial communities, soil enzyme activities, and soil nutrients.

Organic matter selection is crucial for disease control. Here, we hypothesized that (i) the effect of RSD incorporated with liquid easily degradable compounds and solid agricultural waste combination on the soil community composition, structure, and diversity is different; (ii) organic materials with different decomposition characteristics have different effects on soil functional components (nutrient cycling and disease inhibition); and (iii) anaerobic environment may indirectly affect plant growth by changing soil microecological environment. As a means of testing these hypotheses, we used dried perilla (PF), alfalfa (MS) (solid agricultural waste), acetic acid (AA), and ethanol (EA) (liquid degradable compounds) as the representatives of organic materials, respectively. Pot experiments with five treatments were conducted in a ginseng monoculture soil seriously infected by diseases. Research was conducted to determine how RSD-related treatment affected soil microbial community diversity, soil enzyme activity, soil nutrient content, and the growth of replanted seedlings.

## MATERIALS AND METHODS

### Soil sampling and experimental design

The soil that was used in this study was collected from Baixi Forest Farm (44˚05′N, 127˚67′E) in Fusong County, Baishan City, Jilin Province, China. The sampling depth was 20–30 cm. The World Reference Base for Soil Resources classified this soil as alfisol (27). The soil has been continuously planted with ginseng for the past 6 years, and soil samples have been collected after the harvest. The soil’s properties were as follows: pH 5.18; electrical conductivity (EC), 175.45 μS·cm^−1^; organic matter (OM), 105.89 g∙kg^−1^; available nitrogen (AN), 189.26 mg∙kg^−1^; available potassium (AK), 0.19 mg∙kg^−1^; and available phosphorus (AP), 9.02 mg∙kg^−1^.

Perilla (*Perilla frutescens* Britt.) and alfalfa (*Medicago sativa* L.) were collected from local forests, and ethanol and acetic acid were purchased from Beijing Chemical Co., Ltd (Beijing, China). These materials are easily and widely available, with perilla and alfalfa being less expensive than ethanol and acetic acid. Prior to starting the experiment, the perilla and alfalfa were crushed (particle size < 5 mm). Pots (15 × 15 × 12 cm, without drainage holes) were filled with 2 kg of soil, and treatments were conducted as follows: (i) CK, un-treated soil (without substrate), (ii) RSD, soil that had been incorporated with 2% perilla [substrate/soil ratio (wt/wt), the same below), (iii) 2% alfalfa, (iv) 1% ethanol, and (v) 0.25% acetic acid, and then irrigated to saturation and covered with white transparent plastic film (thickness 0.04 mm). There were three replications of these treatments, which were established randomly. The amount of organic material added refers to the amount of liquid and solid added in previous studies (17, 21, 28). The plastic films were removed after 21 days of incubation, with the average day and night temperature in the soil at 30°C–40°C measured using a thermometer. After treatment, three soil cores were collected from each replicate and mixed as a biological replicate. The soil samples (five treatments × three replicates) were sieved and divided into two subsamples, one subsample was stored at −20°C for physicochemical, enzyme activities, and DNA analyses, and another subsample was air-dried for seed germination and seedling growth analysis.

Three hundred seeds of ginseng were surface sterilized by soaking them in 0.3% carbendazim for 2 hours before sowing. Ginseng seeds were seeded in RSD-treated soil with 20 seeds per pot of soil, and each treatment was repeated three times. The pots were incubated in the incubator using a completely random grouping design. Based on the plants with new leaves after 15 days, the survival rate of ginseng seedlings was calculated. In order to eliminate the impact of seed differences, ginseng was then thinned to five plants per pot (leaving healthy ginseng seedlings of similar size) and harvested after 5 months. The roots were carefully washed under running water, and then deionized water was used to remove attached soil particles. After dividing the plants into root and shoot subsamples, the length and fresh weight of the ginseng root were measured.

### Soil physicochemical properties’ analysis

Soil pH and EC were measured by a pH meter (PHSJ-3F, Shanghai, China) and conductivity meter (DDS-307A, Shanghai, China) at a soil-water ratio of 1:5 (wt/vol), respectively. Soil OM was measured by the potassium dichromate external heating method (29). AP was extracted with NaHCO_3_ solution, and then molybdenum-antimony colorimetry was performed (24). AN was measured using the alkali-hydrolyzed diffusing method (30). AK was extracted with 1 mol·L^−1^ NH_4_OAc and then determined by inductively coupled plasma optical emission spectrometer (ICO-OES, iCAP 7400 DUO, Thermofisher, USA) (30).

### Soil enzyme activity analysis

The activities of soil sucrase (SC), urease (UE), acid phosphatase (ACP), and catalase (CAT) were measured using a kit produced by the company Solarbio (Shanghai, China). SC was measured by the colorimetric method of 3, 5-dinitrosalicylic acid, and the activity was defined as 1 mg of reducing sugar produced per gram of soil per day at 37 °C. UE was measured by indophenol blue colorimetry, and the activity was defined as 1 µg NH_3_-N produced per gram of soil per day. ACP was measured by the disodium phenyl phosphate colorimetry, and the activity was defined as 1 nmol phenol release per gram of soil per day at 37°C as one enzyme activity. CAT was measured by colorimetric method, and the activity was defined as catalytic degradation of 1 µmol H_2_O_2_ per gram of air-dried soil sample per day.

### Soil DNA extraction and PCR amplification

Total DNA was extracted from 0.5 g of soil samples using an E.Z.N.A. Soil DNA Kit. We used a NanoDrop 2000 spectrophotometer after extracting DNA to determine its quality and concentration. Agarose gel electrophoresis was used to validate the DNA’s integrity. The 16S rRNA gene V4-V5 and ITS region were used to determine bacteria and fungi abundances, respectively. Polymerase chain reaction (PCR) was conducted using primers 338F/806R and ITS1F/ITS2R for amplification. The protocols described by Zhan et al. (31) and Tan et al. (24) were used for the amplification of bacterial 16S rRNA and fungal ITS genes and analysis of PCR product purity.

### Miseq sequencing and data processing

The diversity and composition of the microbial community were measured using the Illumina Miseq PE300 platform (Illumina, USA) after purification. High-throughput sequencing results have been uploaded to NCBI (SRA bacterial accession number: PRJNA886401; fungal accession number: PRJNA886407). FLASH (version 1.2.11) was used to merge raw sequences generated by MiSeq paired-end sequencing. UPARSE (version 11) was used to cluster quality-filtered bacterial and fungal sequences into operational taxonomic units (OTUs) with 97% sequence similarity, respectively. Representative sequences were taxonomically classified using the Ribosomal Database Project and then according to the Silva database (16S rRNA, version 138) and Unite database (internal transcribed spacers (ITS), version 8.0), with a confidence threshold of 70% for both bacteria and fungi.

### Microbial functional predictions and data analysis

Bacterial and fungal communities were predicted using the FAPROTAX and FUNGuild databases. To avoid overinterpreting fungal functional groups, only functional groups with “probable” and “highly probable” confidence levels were retained, and functional groups with “possible” confidence levels were deleted. Principal coordinate analysis was used to compare microbial communities and functional dissimilarities among treatments based on the Bray-Curtis distance. Linear discriminant analysis (LDA) effect size (LEfSe) was used to identify taxonomic microbial taxa among different treatments. The significance level of microbials at the taxonomic level was LDA > 4 and *P* < 0.05. Microbial networks were constructed using Cytoscape (Version 3.91) software and visualized using Gephi (Version 0.92). Correlation coefficients |*r*| < 0.6 and *P* > 0.05 of the correlation *R* matrix were removed. Heat map correlation analysis was used to visualize the relationships between dominant genera, microbial functions, and environmental factors.

Experimental data were organized using Microsoft Excel. IBM SPSS 21.0 (SPSS Inc., USA) and R (Version 4.1.2) were used to perform all statistical analyses. CK treatment and RSD-related treatment differences were compared using Fisher’s LSD *post hoc* test. GraphPad Prism (Version 8.01) and Majorbio platform are used to create all graphics.

## RESULTS

### Soil physicochemical properties

After anaerobic treatment, soil pH was significantly (*P* < 0.05) higher in all RSD-related treatments than in the CK treatment, with MS treatment having the highest soil pH (Table 1). Soil EC was significantly (*P* < 0.05) lower in all RSD-related treatments than in the CK treatment, and the soil EC of PF treatment was the lowest, but there was no significant difference between EA and MS treatments (Table 1). Compared to the CK treatment, the soil OM content was significantly (*P* < 0.05) increased in PF, MS, and EA treatments, whereas the soil OM content of AA treatment significantly decreased (Table 1). Soil AN content was significantly (*P* < 0.05) higher in all RSD-related treatments than in the CK treatment, and the soil AN content of MS treatment was the highest, whereas there were no significant differences among the PF, EA, and AA treatments (Table 1). Soil AK content in MS and PF treatments was significantly (*P* < 0.05) higher than that in the CK treatment, while it was increased slightly in the EA and AA treatments, with no significant differences compared to the CK treatment (Table 1). Compared to the CK treatment, the soil AP content was significantly (*P* < 0.05) increased in the PF treatment, whereas the soil AP content of MS, EA, and AA treatments significantly decreased (Table 1).

**TABLE 1:** Soil physicochemical properties under different treatments^*a*^

Treatment	pH	EC (μS·cm^−1^)	OM (g∙kg^−1^）	AN (mg∙kg^−1^）	AK (mg∙kg^−1^）	AP (mg∙kg^−1^）
CK	5.21 ± 0.06 e	172.13 ± 2.26 a	107.77 ± 3.97 b,c	189.13 ± 7.75 c	0.209 ± 0.00 c	9.11 ± 0.03 b
PF	5.78 ± 0.03 c	99.63 ± 0.38 d	169.33 ± 3.79 a	243.60 ± 12.12 b	1.144 ± 0.02 b	12.90 ± 0.17 a
MS	6.03 ± 0.01 a	129.67 ± 5.13 c	117.63 ± 15.34 b	576.10 ± 42.58 a	2.291 ± 0.01 a	7.70 ± 0.03 d
EA	5.33 ± 0.02 d	124.77 ± 0.15 c	120.38 ± 3.58 b	238.70 ± 25.93 b	0.226 ± 0.01 c	7.35 ± 0.10 e
AA	5.96 ± 0.01 b	157.93 ± 1.63 b	102.18 ± 3.72 c	265.53 ± 8.35 b	0.210 ± 0.00 c	8.24 ± 0.10 c

^*a*^ The Fisher’s LSD *post hoc* test indicates significant differences at *P* < 0.05 between values (mean ±SD, *n* = 3) within the same column followed by different letters.

### Soil enzyme activity

After anaerobic treatment, soil UE activity in PF, MS, and EA treatments was significantly (*P* < 0.05) higher than that in the CK treatment, while it was increased slightly in the AA treatment, with no significant differences compared to the CK treatment (Table 2). Soil SC activity was significantly (*P* < 0.05) higher in RSD-related treatments than in the CK treatment, with the AA treatment having the highest soil SC activity (Table 2). Compared to the CK treatment, soil ACP activity was significantly (*P* < 0.05) increased in MS, EA, and AA treatments, whereas soil ACP activity of PF treatment significantly decreased (Table 2). The soil CAT activity was significantly (*P* < 0.05) higher in MS and AA treatments (*P* < 0.05) than in CK treatment, while it was increased slightly in the PF and EA treatments, with no significant differences compared to the CK treatment (Table 2).

**TABLE 2:** Soil enzyme activity under different treatments^*a*^

Treatment	UE (U∙g^−1^)	SC (U∙g^−1^)	ACP (nmol∙d∙g^−1^)	CAT (μmol∙d∙g^−1^)
CK	2,947.44 ± 55.51 c	6.59 ± 0.09 e	45,677.53 ± 1,515.77 d	9.46 ± 0.60 c
PF	4,080.47 ± 88.17 b	38.41 ± 1.58 b	32,964.68 ± 891.61 e	11.87 ± 0.15 c
MS	25,428.36 ± 609.85 a	26.17 ± 1.35 c	91,508.08 ± 534.72 a	61.49 ± 3.04 a
EA	3,562.86 ± 126.38 b	24.05 ± 1.02 d	62,978.48 ± 303.99 b	9.60 ± 0.73 c
AA	2,914.03 ± 35.14 c	108.15 ± 0.39 a	61,273.52 ± 181.85 c	22.33 ± 1.40 b

^*a*^ The Fisher’s LSD *post hoc* test indicates significant differences at *P* < 0.05 between values (mean ±SD, *n* = 3) within the same column followed by different letters.

### Soil microbial community and functional diversities

We generated 1,755,718 high-quality bacterial 16S rRNA gene sequences and 1,636,295 high-quality fungal ITS sequences from 15 soil samples in five different treatments using Miseq sequencing. The sequences were clustered into 16,422 and 3981 OTUs with 97% sequence similarity to bacteria and fungi, respectively.

Overall, all RSD treatments had a significant (*P* < 0.05) impact on both bacterial and fungal diversity indices (Table 3). Bacterial richness, diversity, and evenness index were higher in the PF treatment than in the other treatments, and the richness and diversity indexes in the PF treatment were significantly (*P* < 0.05) higher than those in the CK treatment, but there were no significant differences in the evenness index (Table 3). In contrast, the fungal richness index in all RSD treatments was significantly (*P* < 0.05) lower than that in the CK treatment, and the lowest richness index was found in the MS treatment, but the diversity and evenness indexes in the EA treatment were significantly (*P* < 0.05) higher than those in CK treatment (Table 3).

**TABLE 3:** Soil microbial richness, diversity, and evenness under different treatments^*a*^

Treatment	Bacteria			Fungi		
	Sobs	Shannon	Shannoneven	Sobs	Shannon	Shannoneven
CK	851.67 ± 16.29 b	4.44 ± 0.06 b,c	0.66 ± 0.01 a	221.33 ± 5.51 a	2.20 ± 0.13 b	0.41 ± 0.02 b
PF	1,243.30 ± 18.45 a	5.17 ± 0.14 a	0.73 ± 0.02 a	185.00 ± 3.00 b	1.60 ± 0.23 d	0.31 ± 0.04 c
MS	645.33 ± 5.13 c	4.61 ± 0.04 b	0.71 ± 0.01 a	107.00 ± 6.25 c	1.88 ± 0.10 c	0.40 ± 0.02 b
EA	830.33 ± 30.75 b	4.36 ± 0.01 c	0.65 ± 0.01 b	183.67 ± 15.70 b	2.49 ± 0.07 a	0.48 ± 0.01 a
AA	618.33 ± 51.21 c	3.66 ± 0.16 d	0.57 ± 0.02 c	172.67 ± 26.10 b	2.21 ± 0.10 b	0.43 ± 0.01 b

^*a*^ The Fisher’s LSD *post hoc* test indicates significant differences at *P* < 0.05 between values (mean ±SD, *n* = 3) within the same column followed by different letters. The Sobs index represents microbial richness, Shannon index represents microbial diversity, and Shannoneven index represents microbial evenness.

**Fig 1:**
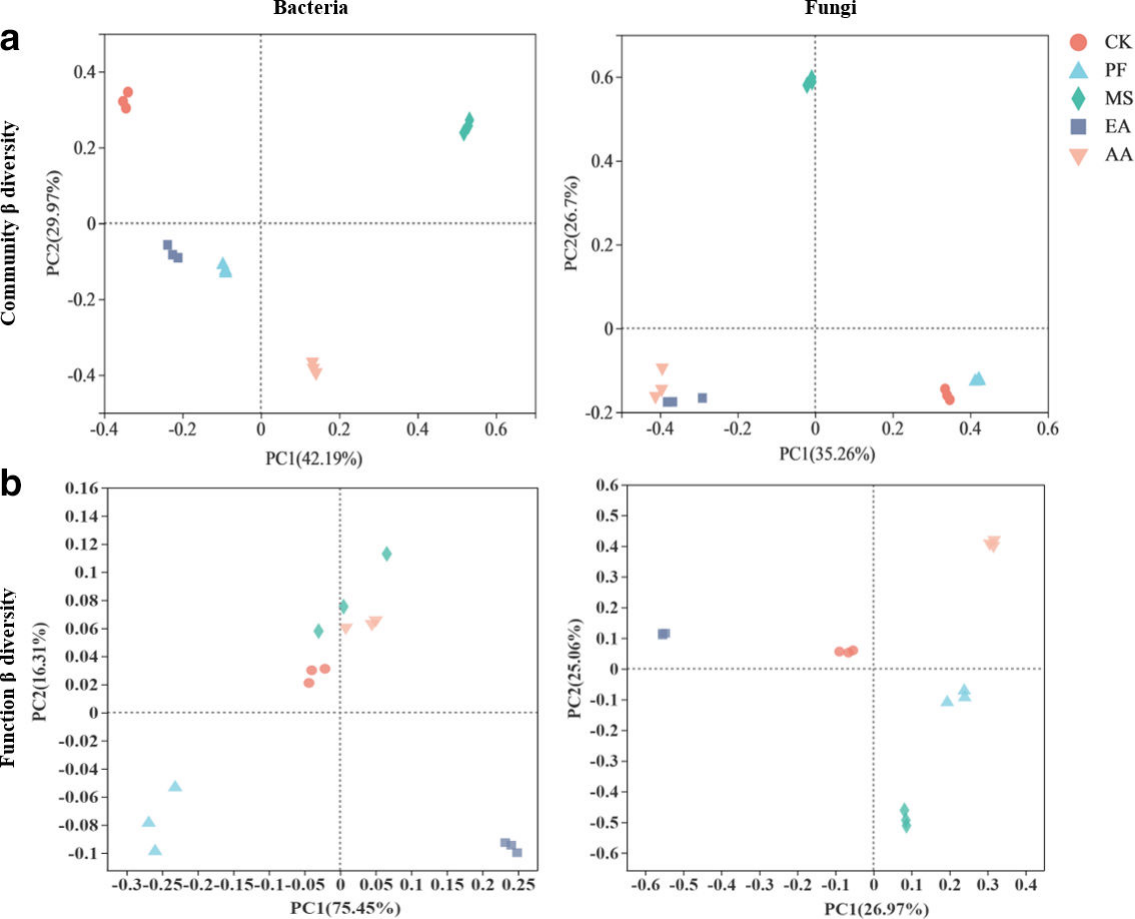
Principal coordinate analysis of the microbial community (a) and functions (b) based on the Bray-Curtis distance in the soils under different treatments.

Similarly, RSD treatments significantly altered the microbial community and functions, according to principal coordinate analysis (Fig. 1). The bacterial community structures in soil treated with PF, EA, and AA were grouped together and separated from soil treated with MS, but the fungal community structures in soil treated with EA and AA were grouped together and separated from soil treated with MS and PF (Fig. 1a). The potential function of bacteria in soil treated with MS and AA was grouped together and separated from soil treated with PF and EA, but the potential function of fungi in soil treated with PF and MS was grouped together and separated from soil treated with EA and AA (Fig. 1b).

### Soil microbial community compositions and potential function prediction

**Fig 2:**
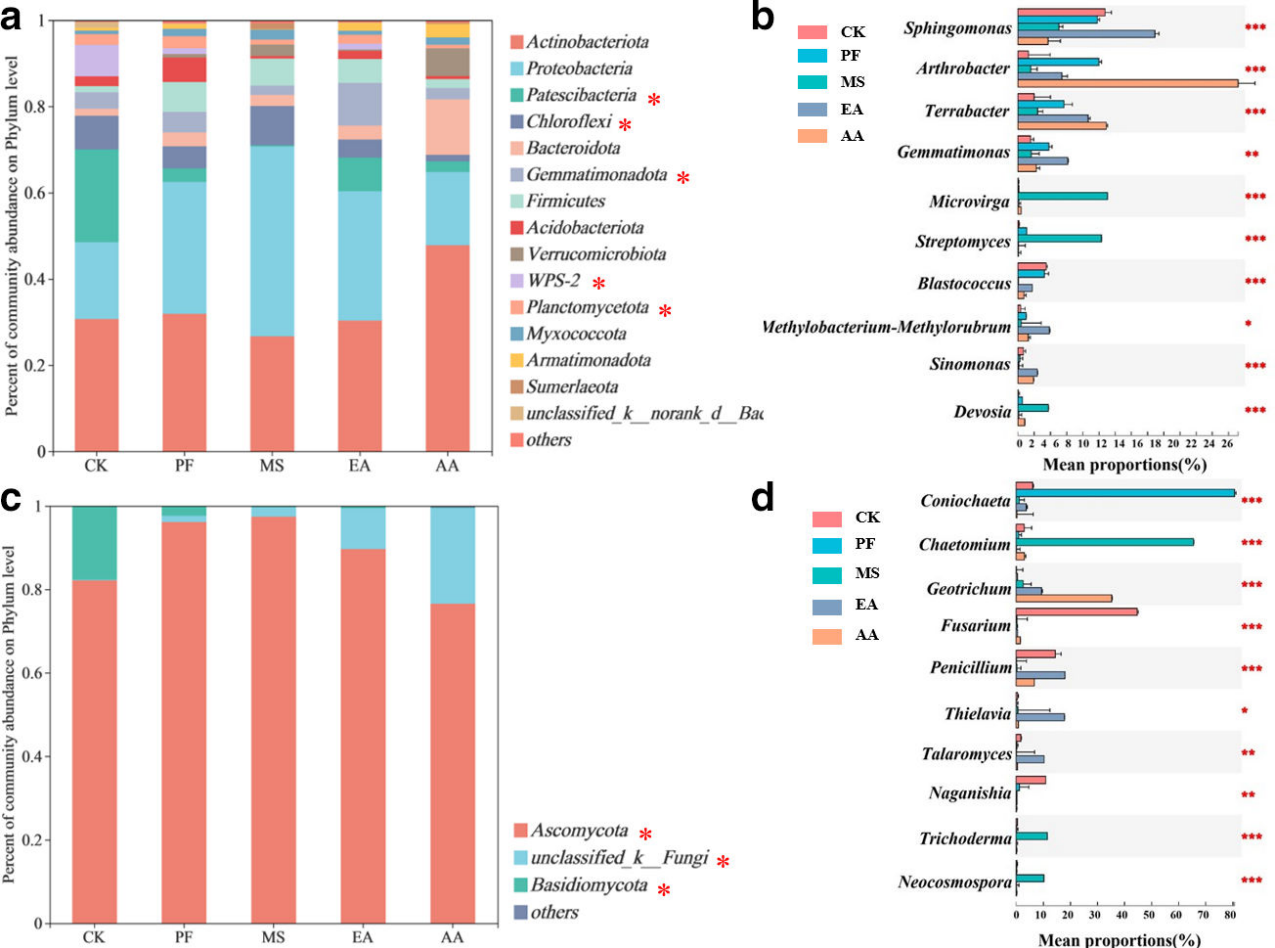
Community composition of dominant bacterial phyla (a) and fungal phyla (c) and relative abundance of the top 10 bacterial (b) and fungal (d) genera in soil samples from different treatments. The bacterial and fungal phyla with relative abundance lower than 1% in all the soils were copolymerized in “Other.” * behind the taxa indicates significant differences at *P* < 0.05 according to Fisher’s LSD *post hoc* test. *, **, and *** behind the taxa indicate significant differences at *P* < 0.05, *P* < 0.01, and *P* < 0.001 according to the one-way ANOVA, respectively.

RSD-related treatments significantly modulated the compositions of soil bacterial and fungal communities, with modulations varying between bacterial and fungal communities (Fig. 2). *Actinobacteriota* and *Proteobacteria* were the dominant bacterial phyla across all soil samples. In four treatments, more than 45% of the sequences classified belong to these phyla (Fig. 2a). *Ascomycota* was the predominant fungal phylum in all the soil samples accounting for more than 75% of the total sequences across all the soils (Fig. 2c). In general, the bacterial communities of the five soils were similar at the genus level, but some specific genera had different relative abundances (Fig. 2b). For instance, the relative abundance of *Arthrobacter*, *Terrabacter*, and *Gemmatimonas* was significantly increased by RSD-related treatments. Obviously, the soil fungal community responded to RSD-related treatments more strongly than the bacterial community (Fig. 2d). In RSD-treated soil, fungal communities differed both in composition and relative abundance of dominant fungal species from those in CK treatments. Specifically, the relative abundance of *Fusarium* decreased significantly in the RSD-related treatments and was only 1.33%–11.48% of that in the CK treatment. *Naganishia* and *Blastococcus* responded similarly to RSD-treated treatments as *Fusarium*.

**Fig 3:**
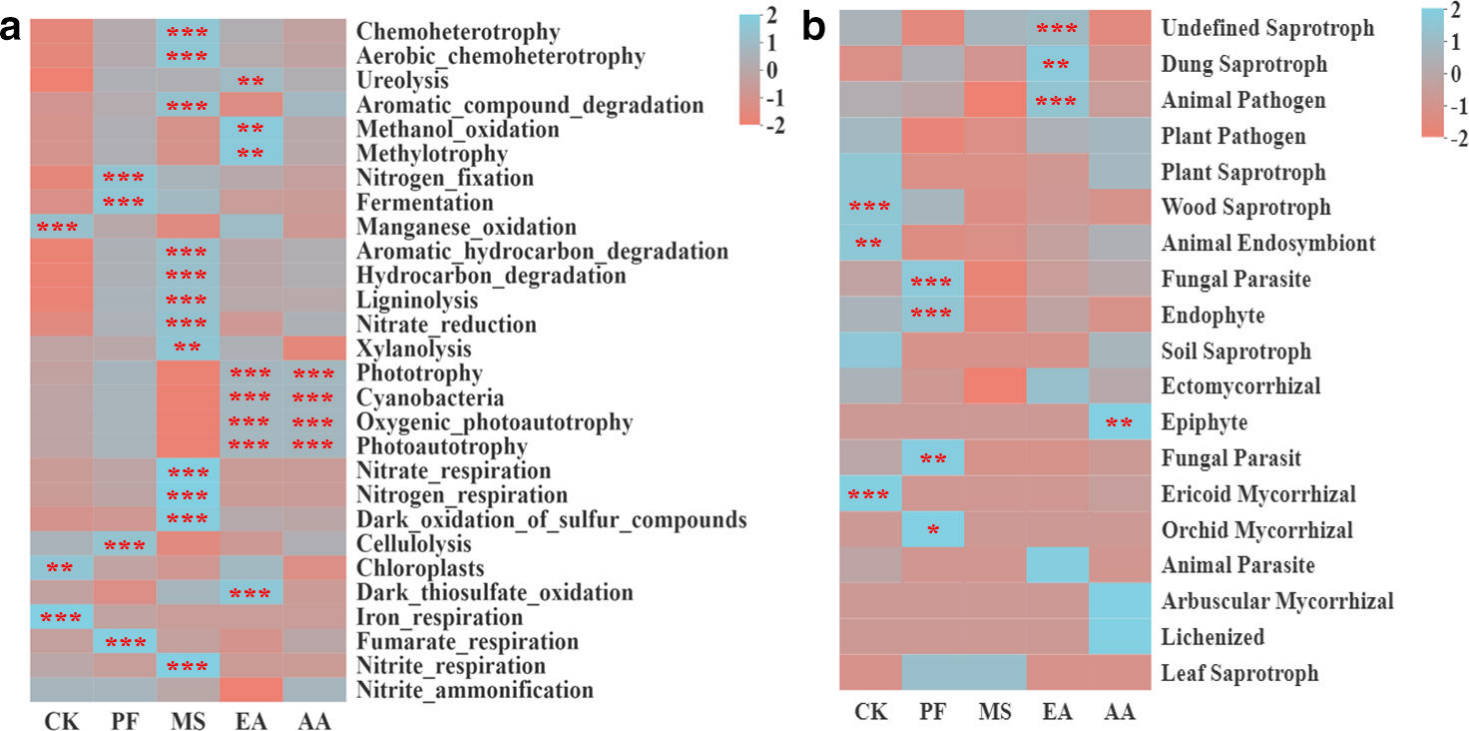
Heatmap displaying the relative abundances of functional groups in bacteria (a) and fungi (b). *, **, and *** behind the taxa indicate significant differences at *P* < 0.05, *P* < 0.01, and *P* < 0.001, red and blue colors indicate the abundance of functional groups in the sample, respectively.

Meanwhile, RSD treatment had a more substantial effect on bacterial functional groups than on fungal functional groups. We found 40 bacterial functional groups and 3 fungal trophic modes (pathotrophs, symbiotrophs, and saprophytes). In RSD-treated soil, the relative abundances of bacterial functional groups related to carbon (C), nitrogen (N), sulfur (S), and hydrogen (H) cycling, such as ureolysis, aromatic compound degradation, nitrogen fixation, hydrocarbon degradation, nitrate reduction, nitrate respiration, dark oxidation of sulfur compounds, dark thiosulfate oxidation, nitrite respiration, and fungal functional groups associated with dung saprotrophy, endophyte, and undefined saprotroph were significantly enriched, compared to CK treatment (Fig. 3a and b). Notably, the relative abundances of bacterial manganese oxidation, iron respiration, fungal plant pathogens, plant saprotroph, animal endosymbiont, and soil saprotroph were decreased in RSD-treated soil (Fig. 3a and b).

### Microbial biomarker taxa and networks’ analysis

**Fig 4:**
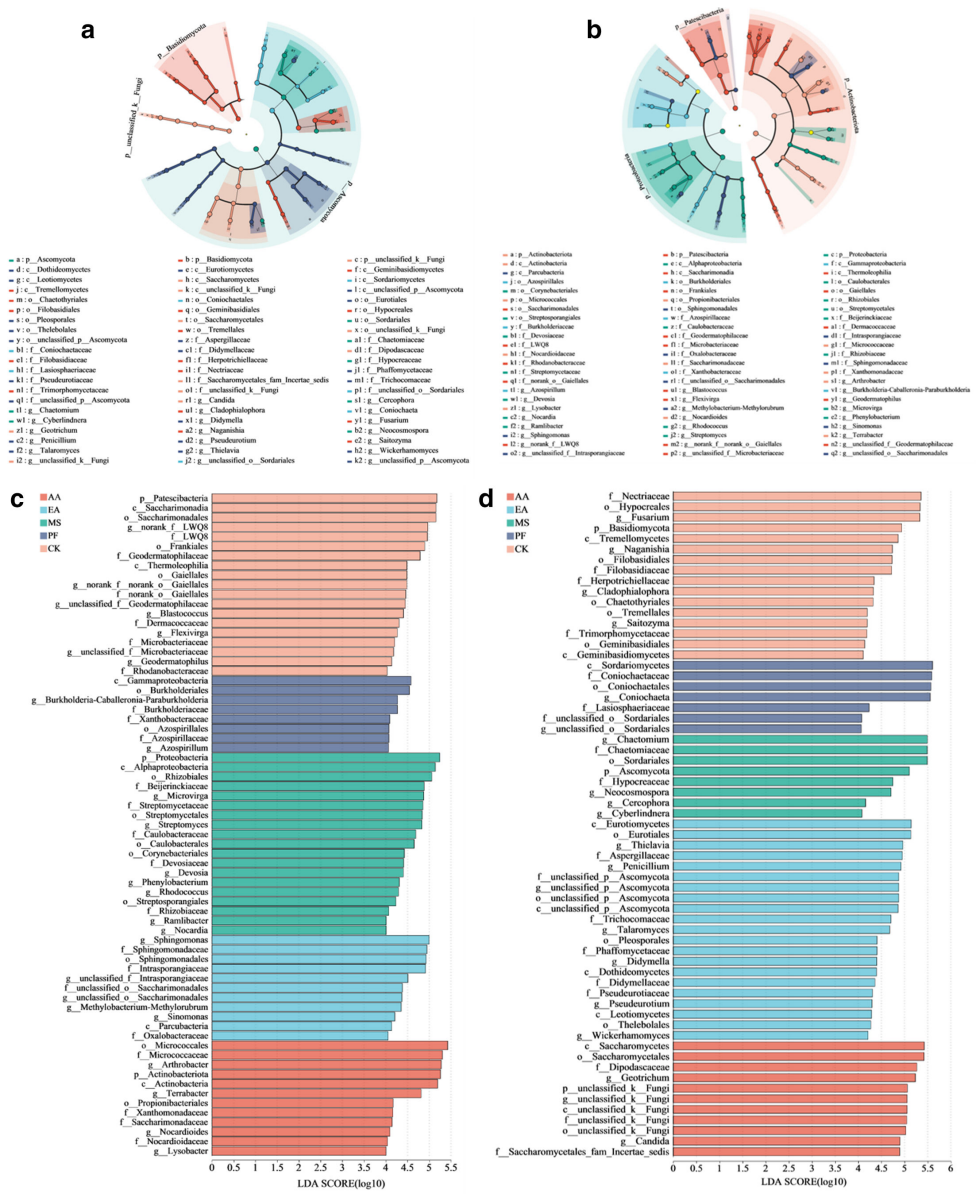
Linear discriminant analysis shows the differences of bacterial taxa (a) and fungal taxa (b) (from phylum to genus) among different treatments, and the significantly enriched bacterial taxa (c) and fungal taxa (d) among different treatments. Taxa with significant differences in abundance (LDA score > 4, *P* < 0.05) between different treatments are colored.

LEfSe analysis revealed that RSD treatments significantly altered bacterial communities from the phyla to the genus level, and that each RSD treatment harbored distinct biomarkers (Fig. 4). For instance, the taxa *Gammaproteobacteria*, *Burkholderiales*, *Xanthobacteraceae*, *Azospirillales*, *Azospirillum*, *Sordariomycetes*, *Coniochaetaceae*, and *Lasiosphaeriaceae* were significantly enriched in PF soil, *Proteobacteria*, *Alphaproteobacteria*, *Beijerinckiaceae*, *Caulobacteraceae*, *Corynebacteriales*, *Streptomyces*, *Phenylobacterium*, *Rhizobiaceae*, *Rhodococcus*, *Streptosporangiales*, *Chaetomiaceae*, *Sordariales*, *Ascomycota*, *Hypocreaceae*, *Microvirga*, *Nocardia*, *Trichoderma*, *Rhizobiales*, and *Neocosmospora* were significantly enriched in MS soil, *Sphingomonas*, *Intrasporangiaceae*, *Sinomonas*, *Parcubacteria*, *Oxalobacteraceae*, *Eurotiales*, *Phaffomycetaceae*, *Aspergillaceae*, *Penicillium*, *Talaromyces*, *Trichocomaceae*, *Pleosporales*, *Dothideomycetes*, *Leotiomycetes*, *Thielavia*, and *Didymella* were significantly enriched in EA soil, whereas *Micrococcaceae*, *Arthrobacter*, *Actinobacteria*, *Propionibacteriales*, *Xanthomonadaceae*, *Terrabacter*, *Geotrichum*, *Nocardioides*, *Nocardioidaceae*, *Saccharomycetes*, *Psathyrellaceae*, *Lysobacter*, and *Candida* were considerably enriched in AA soil (Fig. 4c and d).

**Fig 5:**
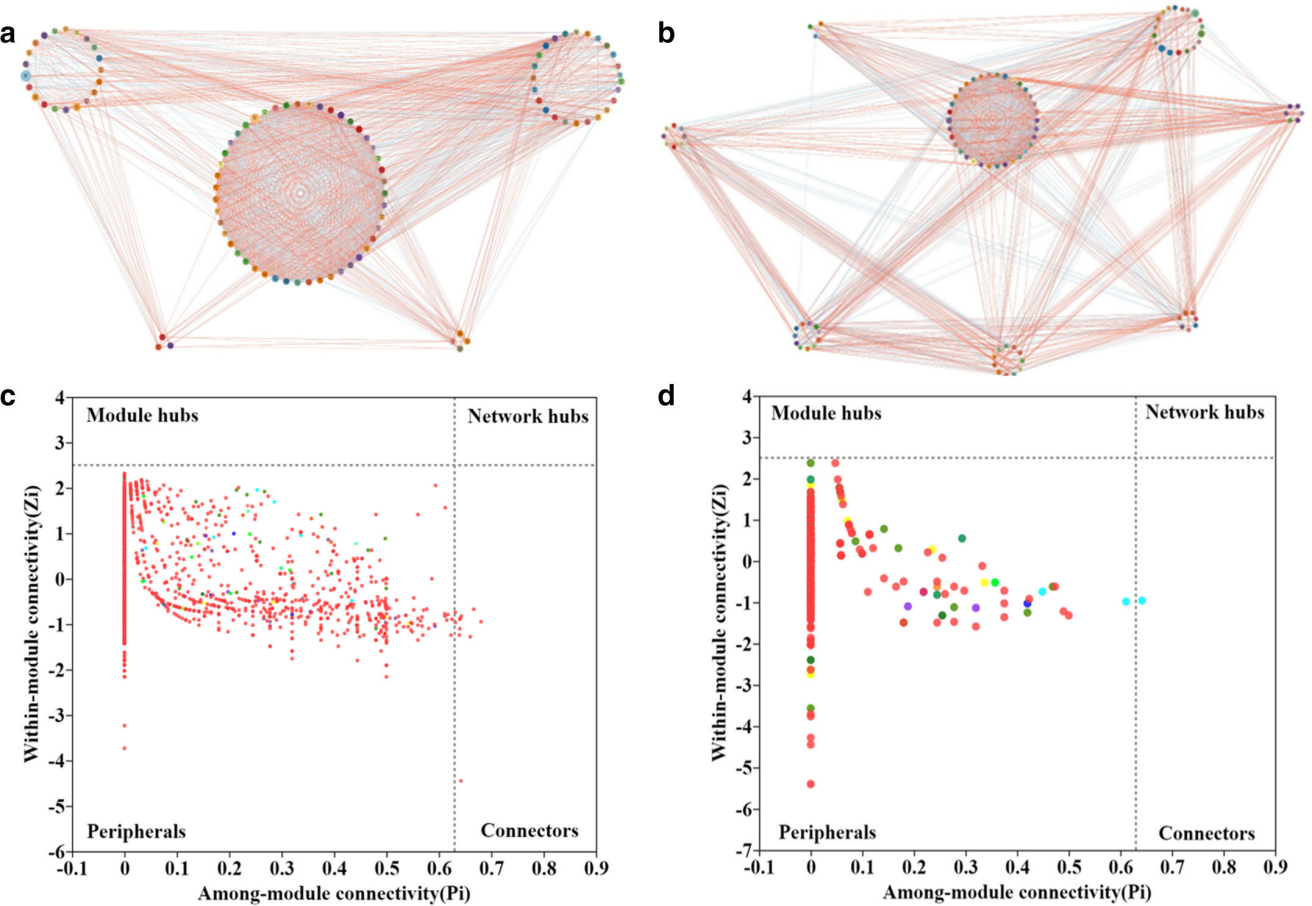
Visualized network of bacterial (a) and fungal (b) related networks for different treatments. The Zi-Pi plot shows the distribution of OTUs of bacteria (c) and fungi (d) based on their topological roles with different treatments. The blue and red lines indicate the negative and positive correlations, respectively.

We found that there were noticeable differences between CK and RSD-related treatments in the bacterial and fungal community networks and their topological characteristics (Fig. 5; Table 4). In the correlation network study, bacteria were grouped into five clusters, while fungi were grouped into eight clusters, indicating stronger interactions between fungal communities than bacterial communities (Fig. 5a and b). Meanwhile, in RSD-related soil, the number of nodes and edges, network heterogeneity, and network centralization of bacterial networks were greater in PF soil, while the number of nodes and edges, characteristic path length, and network heterogeneity of fungal networks were lessened in all RSD-related treatments (Table 4). In the co-occurrence networks, nodes in the regions of connectors (0.24%), module hubs (0.65%), and network hubs (0%) were all identified as keystone species because they all had Zi 2.5 or Pi 0.62 (Fig. 5c and d).

**TABLE 4:** Topological characteristics of microbial community networks under different treatments

Topological characteristics	Bacteria					Fungi				
	CK	PF	MS	EA	AA	CK	PF	MS	EA	AA
Number of nodes	1,131	1,544	828	1,149	837	347	261	154	289	291
Number of edges	2,555	3,730	1,936	2,491	1,855	664	555	321	551	518
Characteristic path length	2.163	2.098	2.128	2.210	2.174	2.361	2.209	2.219	2.350	2.433
Network density	0.004	0.003	0.006	0.004	0.005	0.011	0.016	0.027	0.013	0.012
Network heterogeneity	9.673	11.312	8.264	9.753	8.328	5.314	4.588	3.488	4.849	4.892
Network centralization	0.761	0.814	0.783	0.739	0.800	0.643	0.712	0.727	0.672	0.682
Topological coefficient	0.554	0.477	0.569	0.598	0.671	0.389	0.459	0.661	0.476	0.465

### Relationships between soil properties, enzyme activity, functionality and microbiomes

**Fig 6:**
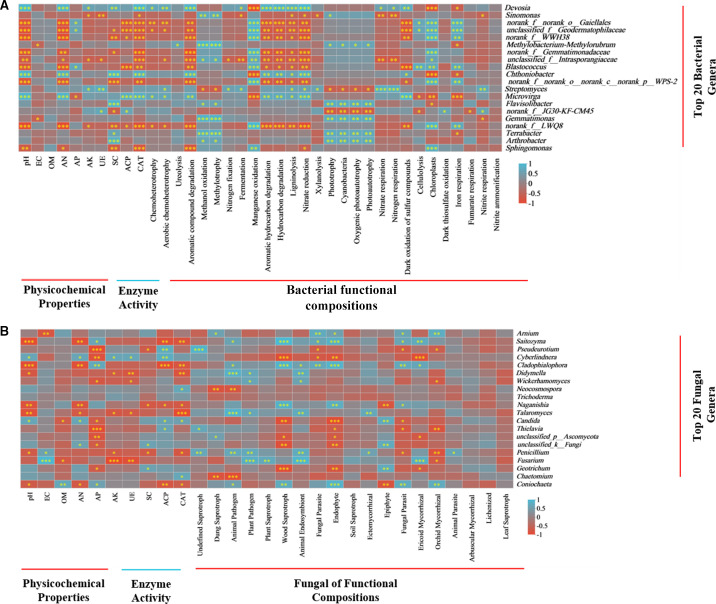
Correlations between dominant genera’ relative abundance and soil properties, enzyme activity, and microbial functions. Relationships between bacterial (a) and fungal (b) relative abundances of dominant genera and soil properties, enzyme activity, and microbial functions. *, **, and *** behind the taxa indicate significant differences at *P* < 0.05, *P* < 0.01, and *P* < 0.001, and the red and blue colors indicate negative and positive correlations, respectively.

The relative abundance of the most dominant genera in RSD-treated soil was significantly (*P* < 0.05) correlated with soil properties, enzyme activity, and functional groups (Fig. 6). For bacteria, the relative abundances of *Devosia*, *Chthoniobacter*, and *Microvirga* were significantly (*P* < 0.05) positively correlated with pH, AN content, CAT activity, aromatic compound degradation, aromatic hydrocarbon degradation, hydrocarbon degradation, ligninolysis, nitrate reduction, and dark oxidation of sulfur compounds, and negatively correlated with manganese oxidation, chloroplasts, and iron respiration (Fig. 6a). *Blastococcus* and *Sphingomonas* relative abundances were significantly (*P* < 0.05) positively correlated with manganese oxidation and chloroplasts, and negatively correlated with pH, AN content, CAT activity, aromatic compound degradation, and nitrate reduction (Fig. 6a). *Sinomonas* and *Gemmatimonas* relative abundances were significantly (*P* < 0.05) positively correlated with methanol oxidation, methylotrophy, and phototrophy but negatively correlated with nitrite respiration (Fig. 6a). However, *Flavisolibacter*, *Terrabacter,* and *Arthrobacter* relative abundances were significantly (*P* < 0.05) positively correlated with SC activity, methanol oxidation, methylotrophy, phototrophy, cyanobacteria, oxygenic photoautotrophy, and photoautotrophy (Fig. 6a).

For fungi, *Saitozyma*, *Cladophialophora*, and *Coniochaeta* relative abundances were significantly (*P* < 0.05) positively correlated with AP content, animal pathogen, wood saprotroph, endophyte, and fungal parasite, and negatively correlated with AN content, ACP activity, and CAT activity (Fig. 6b). *Didymella*, *Talaromyces*, and *Penicillium* relative abundances were significantly (*P* < 0.05) positively correlated with animal pathogen, plant pathogen, and animal endosymbiont, and negatively correlated with pH, AK content, and CAT activity (Fig. 6b). *Pseudeurotium* and *Thielavia* relative abundances were significantly (*P* < 0.05) positively correlated with ACP activity and undefined saprotroph, and negatively correlated with AP content, fungal parasite, and orchid mycorrhiza (Fig. 6b). *Chaetomium* and *Neocosmospora* relative abundances were significantly (*P* < 0.05) positively correlated with CAT activity, and negatively correlated with dung saprotroph and animal pathogen (Fig. 6b). *Candida* and *Cyberlindnera* relative abundances were significantly (*P* < 0.05) positively correlated with pH, AN content, and ACP activity, and negatively correlated with AP content and wood saprotroph (Fig. 6b). Additionally, *Fusarium* relative abundances were significantly (*P* < 0.05) positively correlated with EC, plant pathogen, plant saprotroph, animal endosymbiont, and ericoid mycorrhiza, and negatively correlated with OM content, AN content, UE activity, and orchid mycorrhiza (Fig. 6b).

### Seed germination and seedling growth

**Fig 7:**
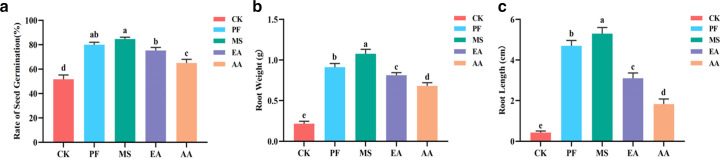
Seed germination and seedling growth under different treatments. The error bars indicate the standard errors of the means of three replicates. The Fisher’s LSD *post hoc* test indicates that the letters denote a significant difference at *P* < 0.05. (a) represents the germination rate of seeds, (b) represents root weight, (c) represents root length.

RSD-related treatment significantly (*P* < 0.05) promoted ginseng seed germination and seedling growth. The MS treatment had the highest seed germination rate, root weight, and root length, which were 1.64 times, 4.97 times, and 12.42 times that of the CK treatment, respectively (Fig. 7). At the same time, ginseng’s germination rate, root weight, and root length were MS > PF > EA >AA, which indicated that the combination of reductive soil disinfestation and solid agricultural waste had a stronger effect on ginseng seed germination and seedling growth than the combination of reductive soil disinfestation and liquid easily degradable compounds (Fig. 7).

## DISCUSSION

### RSD incorporated with solid agricultural waste can reorganize soil microbial communities and inhibit soilborne pathogens’ growth

The soil microbial community is essential for nutrient cycling, plant growth, and disease prevention and control (13, 32). Numerous studies have shown that RSD treatment significantly changes soil microbial communities and helps to resist pathogen invasion (10, 33). In agreement with these studies, we found that RSD treatment significantly decreased the relative abundance of known soilborne pathogen *Fusarium* and increased the relative abundance of known disease-suppressive agents *Arthrobacter*, *Streptomyces*, *Terrabacter*, and *Gemmatimonas*. Members of the genera *Gemmatimonas* and *Arthrobacter*, for example, inhibit pathogenic microorganism growth by producing lipopeptides and bacteriocins (34, 35). Meanwhile, *Streptomyces* has the ability to degrade autotoxic substances and has been found to successfully control *Fusarium oxysporum* and *Botrytis cinerea*-caused soilborne diseases (36). Additionally, the type and amount of the organic materials have a greater effect on the generation of anaerobic conditions and the production of volatile organic compounds toxic to plant pathogens (18, 37). For example, RSD incorporated with maize straw can effectively inhibit *Artemisia selengensis* root rot pathogens and the inhibition efficiency can reach 90% when the application rate is 2% (38). Besides, previous studies have found that highly and moderately labile C sources increase the rate of attainment of anaerobicity, a condition that is critical for the success of RSD (32, 39). In this study, perilla and alfalfa were mainly composed of lignin, cellulose, and hemicellulose, which are more difficult to decompose and utilize than ethanol and acetic acid. Therefore, the rest of the C sources would continuously stimulate the aerobic microorganisms and improve soil microbial activity after RSD treatment.

We also observed that PF and MS treatments significantly increased bacterial diversity while decreasing fungal diversity because bacteria were dominated by anaerobic or facultative anaerobic taxa, whereas most fungi lived in aerobic environments (3). Consequently, RSD treatment increased bacteria diversity and richness by occupying ecological niches and inhibiting fungal pathogen activity. Interestingly, EA and AA treatments decreased bacterial diversity but increased fungal diversity. Considering that ethanol and acetic acid are both pure chemicals, it is likely that the microbiome succession was primarily determined by the type of materials used under anaerobic conditions (19). Although RSD treatment with different carbon sources alters microbial community richness, it also cultivates a unique core microbiota. For instance, the taxon *Azospirillum* was significantly enriched in PF soil, the taxon *Chaetomiaceae* was significantly enriched in MS soil, the taxon *Penicillium* was significantly enriched in EA soil, and the taxon *Lysobacter* was significantly enriched in AA soil. Studies have shown that such core microbiomes have a variety of potential beneficial effects in maintaining soil health and plant growth, such as antibiotic production, heterologous material degradation, heavy metal adsorption, and root colonization (35, 40, 41). *Chaetomium* produces cellulase and chaetomin, which can inhibit pathogenic fungi growth and promote the activation of soil nutrients (42, 43). *Penicillium* can degrade harmful substances in soil, and its secondary metabolites can significantly inhibit some pathogens (44).

The microbial networks of healthy soils are thought to be more complex than those of diseased soils, indicating that the microbial network’s characteristics are crucial for predicting plant health status (45, 46). Bacteria were found to be grouped into five clusters in this study, whereas fungi were found to be grouped into eight clusters, indicating stronger interactions between fungal communities than bacterial communities. Interestingly, the number of nodes and edges, characteristic path length, and network heterogeneity of fungal networks were lessened in all RSD-related treatments. This is because the presence of antifungal compounds resulting from the decomposition of various organic substrates during RSD treatment may inhibit fungal taxa growth (33, 47).

### RSD incorporated with solid agricultural waste combination can restore degraded soil and improve soil functions

RSD had effects on soil properties, enzyme activities, and functions in addition to improving the microbial community and resisting soilborne pathogens invasion (3, 14). Acidification and salinization are two major characteristics of soil degradation (3). The EC value, which is a common indicator of soil salinity in the current study, was significantly reduced in RSD-related treatments, with the lowest value observed in the PF treatment, which is consistent with previous reports (14). Similarly, pH values of acidified soils were generally increased after RSD treatment, which was consistent with our results, resulting from the consumption of H^+^ through denitrification and other redox reactions under anaerobic conditions (48). Additionally, decomposable carbon sources and irrigation affect enzyme activities and macronutrients, and their transformation varies among different studies. We found that the contents of OM, AN, and AK and the activities of UE, SC, and ACP were increased in PF and MS treatments, which may be directly due to the anaerobic degradation of perilla and alfalfa, or indirectly due to the enhancement of nutrient cycling.

Soil functional diversity plays a crucial role in microbial behavior (18). We found that RSD treatment affected bacterial functional groups more significantly than fungal functional groups. This may be because the bacterial microbiota is strongly stimulated during the decomposition of organic materials, which leads to an increase in functional diversity and is consistent with our observations that RSD-related treatments more strongly favored bacterial community diversity (33, 49). RSD-related treatments also increased microbial functions related to nutrient cycling, endophytes, and dung saprotrophs while decreasing fungal plant pathogens. The previously reported increase in OM, AN, and AK contents, as well as UE, SC, and ACP activities during RSD treatment, may be attributable to these increased nutrient cycling functions, which include ureolysis, nitrogen fixation, nitrate reduction, nitrogen respiration, and aromatic compound degradation (50, 51).

### Linking reassembled soil microbiomes to soil properties, enzyme activities, and microbial functions

The soil’s abiotic environment is critical for reorganizing the microbial community, improving the soil environment, and promoting seedling growth (13). Studies suggested that the primary variables influencing soil microbial communities are EC, pH, OM, UE, SC, and ACP (3, 34). Soil EC, pH, OM, AN, AK, and AP contents and UE, SC, ACP, and CAT activities were found to be significantly related to microbial taxa in this study. Research has shown that a slight change in soil pH (1.5 units) can have a 50% impact on bacterial activity (13). Soil pH was found to be significantly positively correlated with *Devosia*, *Chthoniobacter*, *Microvirga*, *Candida*, and *Cyberlindnera*, and significantly negatively correlated with *Blastococcus*, *Sphingomonas*, *Didymella*, and *Talaromyces*. These demonstrated that pH was an important factor in microbial community transformation and was strongly related to microbial richness and diversity (52). Fusarium wilt, caused by *Fusarium oxysporum*, is among the most dangerous soilborne diseases and can affect plants of a wide range (53). According to numerous studies, RSD practices can effectively decrease soilborne pathogens (54, 55), which is consistent with our study that *Fusarium* abundance was significantly decreased in all RSD treatments. Similarly, there was a significant positive correlation between soil EC and *Fusarium* abundance, supporting the earlier findings. Interestingly, the decomposability characteristics of added organic materials may jointly determine the degree to which microbial activity can be improved through community changes, as was the case when soil OM was found to be significantly negatively correlated with *Fusarium* (3).

Previous research found that RSD enhances soil nutrient cycling functions by regulating the relative abundance of specific taxa within core microbiomes (3). The majority of the dominant genera that showed a noticeable increase in RSD-treated soils were found to be closely related to the aforementioned nutrient cycling functions, such as *Terrabacter, Flavisolibacter*, and *Arthrobacter,* and methanol oxidation, methylotrophy, phototrophy, cyanobacteria, oxygenic photoautotrophy, and photoautotrophy were positively correlated. *Flavisolibacter* is an important iron-reducing agent, converting Fe^3+^ to Fe^2+^ both directly and indirectly under anaerobic conditions, and Fe^2+^ accumulation significantly inhibits plant pathogen growth (50, 56). *Sphingomonas* is a microorganism that has good environmental adaptability and tolerance and can degrade polycyclic aromatic hydrocarbons and phenols (31). *Sphingomonas* was positively correlated with manganese oxidation and chloroplasts but negatively correlated with aromatic compound degradation or nitrate reduction. In addition, *Devosia*, *Chthoniobacter*, and *Microvirga* have been identified as important decomposers of various C and H compounds, thereby stimulating the soil nutrient cycle.

Maintaining soil health is recognized to be a prerequisite for successful replanting failure alleviation; however, the primary indicator for evaluating soil health is whether replant seedlings survive (9). Our results indicated that RSD-related treatment significantly promoted ginseng seed germination and seedling growth. Consistent with previous studies, the MS treatment had the highest seed germination rate, root weight, and root length, which were 1.64 times, 4.97 times, and 12.42 times higher than the CK treatment, respectively (8). This was most likely due to RSD’s beneficial effects on soil chemical and microbial properties, which reversed some of the previous cropping systems’ negative effects on plant growth. Additionally, the effect of strong reduction soil sterilization combined with solid agricultural waste on ginseng seed germination and seedling growth was stronger than that of strong reduction soil sterilization combined with liquid easily degradable compounds. Differences in the chemistry (like degradability) and quantity (like availability) of carbon sources in the organic materials used could explain this disparity.

### Conclusions

By reorganizing microbial communities and repairing the soil environment, reductive soil disinfestation can significantly reduce plant replant failure in combination with liquid degradable compounds and solid agricultural waste. However, the effect of RSD combined with solid agricultural wastes is better than that of liquid easily degradable compounds. In particular, increased bacterial diversity and abundance of beneficial taxa and decreased fungal diversity and pathogenic taxa rebalance the soil microbiome. Meanwhile, RSD treatment increased microbial functions related to the cycling of carbon, sulfur, nitrogen, and hydrogen while decreasing plant pathogen function. The majority of the dominant genera that increased significantly in RSD-treated soils were closely related to the aforementioned nutrient cycling functions, including *Gemmatimonas*, *Streptomyces*, *Arthrobacter*, and *Terrabacter*, many of which are also known to be disease-suppressive agents. Furthermore, RSD-related treatment also changed soil properties and enzyme activities, especially increased soil pH, AN, AK, S-SC, and S-CAT contents and activities and decreased soil EC values. Importantly, RSD-related treatments also significantly promoted seed germination and seedling growth. Thus, RSD practice using various organic residues may improve soil quality and microbial community structure, inhibit the proliferation of pathogenic bacteria, and contribute to the growth of replanted crops, making it a potential agricultural practice. However, the effect of RSD combined with solid agricultural wastes is better than that of liquid easily degradable compounds, which may be related to the stability and long duration of action of solid agricultural wastes.

## Data Availability

The data sets presented in this study can be found in online repositories. The names of the repository/repositories and accession number(s) can be found at PRJNA886401 and PRJNA886407.
